# Optimization and Characterization of Acetic Acid-Hydrolyzed Cassava Starch Nanoparticles for Enhanced Oil Recovery Applications

**DOI:** 10.3390/polym17081071

**Published:** 2025-04-16

**Authors:** Mohammed E. Ali Mohsin, A. F. A. Rahman, Zakiah Harun, Agus Arsad, Suleiman Mousa, Muhammad Abbas Ahmad Zaini, Mohammad Yousef Younes, Mohammad Faseeulla Khan

**Affiliations:** 1 Department of Chemical Engineering, College of Engineering, King Faisal University, Al Ahsa 31982, Saudi Arabia; saamousa@kfu.edu.sa (S.M.); myounes@kfu.edu.sa (M.Y.Y.); 2 UTM-MPRC Institute for Oil and Gas, Faculty of Chemical and Energy Engineering, Universiti Teknologi Malaysia, 81310 Johor Bahru, Johor, Malaysia; 3 Centre of Lipids Engineering & Applied Research, Ibnu-Sina Institute for Scientific & Industrial Research, Universiti Teknologi Malaysia, 81310 Johor Bahru, Johor, Malaysia; 4 Department of Mechanical Engineering, College of Engineering, King Faisal University, Al-Ahsa 31982, Saudi Arabia

**Keywords:** cassava starch nanoparticles, ultrasonication, acid hydrolysis, response surface methodology, enhanced oil recovery

## Abstract

This study presents an optimized and sustainable route for synthesizing cassava starch nanoparticles (CSNPs) tailored for enhanced oil recovery (EOR) applications. Conventional inorganic acid hydrolysis methods often produce low nanoparticle yields and large particle sizes due to extensive degradation of both amorphous and crystalline starch regions. To overcome these challenges, ultrasonic-assisted acetic acid hydrolysis coupled with response surface methodology (RSM) was applied. Under optimal conditions, two distinct CSNPs were produced: CSNP A (206.77 nm, 96.23% yield in 3 days) and CSNP B (99.4 nm, 96.07% yield in 7 days). Characterization via Fourier transform infrared (FTIR) spectroscopy and X-ray diffraction (XRD) confirmed enhanced crystallinity, while rheological analyses revealed shear-thickening behavior and improved viscosity, key factors for effective polymer flooding in EOR. DSC and TGA measurements highlighted robust thermal stability, essential for high-temperature reservoir conditions. A preliminary assessment suggests CSNP B’s small size (99.4 nm), high viscosity, and thermal stability make it particularly promising for EOR in low-permeability reservoirs, with future core flooding studies needed for validation. These attributes position CSNPs as sustainable alternatives for polymer flooding in challenging reservoir environments.

## 1. Introduction

Enhanced oil recovery (EOR) is an essential method for maximizing crude oil extraction from reservoirs once primary and secondary recovery techniques become insufficient. Among various EOR methods, polymer flooding has gained significant attention due to its ability to increase the viscosity of injected fluids, thereby enhancing oil displacement efficiency by improving the mobility ratio between oil and water phases [1]. Commonly utilized polymers in EOR, such as hydrolyzed polyacrylamide (HPAM) and xanthan gum, face major challenges in high-temperature and high-salinity reservoir conditions, often leading to thermal, mechanical, and microbial degradation [2]. Consequently, there is growing interest in alternative polymers and additives that can maintain their viscosity and structural integrity under such harsh environments.

To address these challenges, researchers have explored the use of inorganic nanoparticles, such as titanium dioxide and silica, as additives to enhance the rheological properties of polymers in EOR applications, leveraging their ability to withstand high-temperature and high-salinity conditions [3]. However, issues like nanoparticle aggregation, poor dispersion, and environmental concerns limit their practical application [4]. Additionally, the need to combat global warming necessitates the pursuit of environmentally efficient solutions. Biopolymers like cassava starch (CS) have emerged as promising, eco-friendly alternatives due to their biodegradability, availability, cost-effectiveness, and excellent swelling capacity in water [5]. Despite these advantages, native cassava starch exhibits limitations, including large particle sizes that can clog reservoir pores and instability in high-temperature and high-salinity conditions [6]. Reducing cassava starch to the nanoscale—resulting in cassava starch nanoparticles (CSNPs)—increases surface area, enhances stability, and improves interactions with reservoir fluids, thereby increasing viscosity and aiding efficient oil recovery [7].

Despite the potential of CSNPs, traditional synthesis methods often face challenges in achieving high yields and stability. For instance, acid hydrolysis with strong inorganic acids like sulfuric acid (H_2_SO_4_) and hydrochloric acid (HCl) has been linked to low yields and lengthy processing times, with Shahrodin et al. reporting yields as low as 0.54% to 1.10% after treating native cassava starch with H_2_SO_4_ at 37 °C for 5 to 7 days [8]. Recent advances in starch modification offer promising alternatives. Alassmy et al. (2023) demonstrated the potential of sustainable organocatalytic esterification to enhance starch stability, using potato starch and acetic anhydride to achieve intermediate degrees of substitution (0.2 < DS < 1.5), offering insights into green chemistry approaches that can be adapted for EOR [9]. Matovanni et al. (2023) synthesized cassava starch-grafted polyacrylamide hydrogel using a microwave-assisted method, achieving high viscosity and stability under reservoir conditions, directly supporting its potential for EOR polymer flooding [10]. Akinyemi et al. (2021) evaluated cassava starch as a viscosifier in water-based drilling muds, demonstrating its rheological enhancement under oilfield conditions, and suggesting its potential for EOR with further modification [11]. Additionally, Qi et al. (2024) reported that extrusion-induced modification of cassava starch reduces molecular weight and enhances enzymatic hydrolysis, offering a sustainable approach to improve starch functionality that could be adapted for EOR applications [12]. These advancements highlight the potential of modified cassava starch as a sustainable alternative to synthetic polymers in challenging EOR environments.

Ultrasonication has emerged as a promising technique to further enhance the synthesis process by providing mechanical forces that facilitate the dislodging and fragmentation of starch granules, selectively targeting amorphous regions while preserving crystalline domains essential for mechanical stability [13]. This method also reduces particle aggregation, ensuring that the resulting nanoparticles remain well-dispersed, which is crucial for reliable viscosity enhancement and injectivity in reservoir operations. However, systematic studies on the combined and optimized effects of acetic acid hydrolysis and ultrasonication for producing high-performance CSNPs tailored for EOR remain limited.

This study aims to bridge this gap by employing response surface methodology (RSM) to optimize key parameters—acid concentration, temperature, and hydrolysis time—for an ultrasonication-assisted acetic acid synthesis route to CSNP production [14]. The objective is to achieve consistently high yields of small, stable nanoparticles capable of delivering robust viscosity and withstanding the high-salinity, high-temperature environments typical of oil reservoirs. This optimized process will contribute to greener manufacturing practices and offer an alternative to strong-acid hydrolysis processes, supporting efforts to enhance oil recovery through more durable and environmentally responsible polymer flooding agents.

## 2. Experimental

### 2.1. Materials

The cassava starch powder used in this study was reagent-grade (CAS No. 9005-25-8) and obtained from QREC (Asia) Sdn. Bhd. (Selangor, Malaysia). This commercial cassava starch exhibited an amylose-to-amylopectin ratio of approximately 15–25% amylose and 75–85% amylopectin. Glacial acetic acid (CAS No. 64-19-7, 99% purity) used as the organic acid for the hydrolysis process was also supplied by QREC. All chemicals used in this study, including the acetic acid and starch powder, were commercially available and did not require further purification. An ultrasonicator (Elmasonic S 70H, Elma Schmidbauer GmbH, Singen, Germany) and a centrifuge (Eppendorf 5810R, Eppendorf SE, Hamburg, Germany) were employed for the ultrasonication hydrolysis and centrifugation steps. These instruments were carefully calibrated to maintain consistency across experimental runs.

### 2.2. Cassava Starch Nanoparticle (CSNP) Preparation

**Figure 1:**
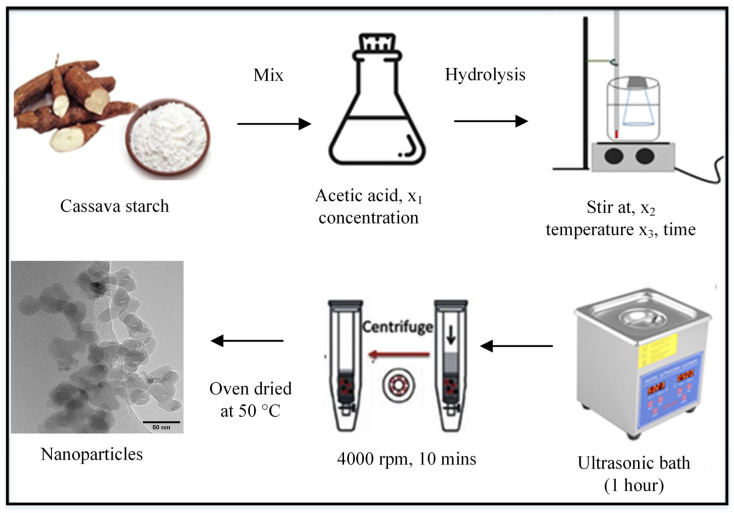
Schematic depiction of the CSNP synthesis, illustrating the acetic acid hydrolysis, neutralization, centrifugation, drying, and final nanoparticle collection steps.

CSNPs were synthesized using ultrasonic-assisted acetic acid hydrolysis. In a typical procedure, 20 g of native cassava starch was mixed with 125 mL of an acetic acid solution of known concentration in a 250 mL Erlenmeyer flask. The mixture was stirred continuously at a controlled speed and temperature for a fixed duration. Following this, the suspension was placed in a high-intensity ultrasonication bath with a frequency of 40 kHz and a power output of 70 W for one hour. The high-intensity ultrasonication was used to reduce the molar mass and prevent aggregation of the nanoparticles [15]. The resulting crude suspension of CSNPs was centrifuged at 4000 rpm for 10 min to separate the nanoparticles from the unreacted materials. The nanoparticles were washed multiple times with deionized water until neutrality was achieved. They were then dried in an oven at 40 °C for 24 h to ensure complete removal of any residual moisture and acid, yielding dry CSNPs suitable for further characterization. The schematic depiction of the CSNP synthesis is illustrated in Figure 1.

The percentage recovery yield of the CSNP was calculated as follows:

(1) $$Recovery\;yield(\%)=\frac{W_{f}}{W_{i}}\times 100\%$$

where *w_f_* is the final weight of starch after oven drying, and *w_i_* is the initial weight of native starch before acid hydrolysis.

### 2.3. Characterization Techniques

The microstructure and surface morphology of CS were characterized using a VP-SEM Zeiss (Model 1450VP, Carl Zeiss AG, Oberkochen, Germany) to observe the particle shape and surface features, providing insights into the granule structure before nanoparticle formation. For the CSNPs, high-resolution transmission electron microscopy (HR-TEM, Model HT7700, Hitachi High-Technologies Corporation, Tokyo, Japan) was used at an accelerating voltage of 120 kV. Dynamic light scattering (DLS) was employed using a Litesizer 500 (Anton Paar GmbH, Graz, Austria) to measure the particle size distribution of both the native CS and CSNPs in their dispersed states. Samples were prepared by dispersing the CS and CSNPs in distilled water at a concentration of 0.1 wt. %, ensuring homogeneity for accurate particle size analysis. Rheological properties were determined using a 350 RST Brookfield Rheometer at a constant temperature of 25 °C to ensure consistency across samples. Thermal properties were assessed through differential scanning calorimetry (DSC) and thermogravimetric analysis (TGA). A DSC instrument (DSC 1, Mettler-Toledo GmbH, Greifensee, Switzerland) was used with a heating rate of 10 °C/min, analyzing temperature ranges from 30 to 300 °C to observe thermal transitions. The thermal stability of the samples was further evaluated using a TGA instrument (TGA 4000, PerkinElmer Inc., Waltham, MA, USA), measuring mass loss from 50 °C to 600 °C under a nitrogen atmosphere. The chemical structure and functional groups present in native CS and CSNPs were analyzed using Fourier transform infrared (FTIR) spectroscopy. Samples were prepared using potassium bromide (KBr) to facilitate infrared transparency. FTIR spectra were collected using an FTIR spectrometer (Spectrum Two, PerkinElmer Inc., Waltham, MA, USA) to identify key functional groups and any changes in chemical bonding resulting from the acid hydrolysis process. The crystalline structure of the starch was analyzed using X-ray diffraction (XRD) with a SmartLab diffractometer (Rigaku Corporation, Tokyo, Japan). The relative crystallinity (RC) was quantitatively determined based on the diffractogram, following the equation:

(2) $$RC\;(\%)=\frac{A_{c}}{A_{a\;}+A_{c}}$$

where *A_c_* represents the crystalline area and *A_a_* is the amorphous area.

### 2.4. Design of Experiment (DoE)

Response surface methodology (RSM) was employed to optimize the independent variables—acid concentration (ϰ_1_), temperature (ϰ_2_), and hydrolysis time (ϰ_3_)—to maximize yield, minimize particle size, and optimize viscosity by adopting a central composite rotatable design (CCRD), as illustrated in Figure S1 (see Supplementary Materials). The experimental ranges and coded levels of the independent variables are shown in Table S1 in the Supplementary Materials. This allowed the determination of two distinct CSNP samples—CSNP A (206.77 nm, 96.23% yield) and CSNP B (99.4 nm, 96.07% yield)—produced under varying conditions of reaction time and temperature. The CCRD matrix (Table S2 in Supplementary Materials) consisted of eight factorial points (trials 1–8), six axial points (trials 9–14), and six replications at the central points (trials 15–20), where all the coordinates were equal to zero. The running order of the trials was randomized to avoid any systematic errors.

## 3. Results

### 3.1. Characterization of CSNPs

#### 3.1.1. Recovery Yield

The yield of cassava starch nanoparticles (CSNPs) after acid hydrolysis was quantified under two optimized conditions, achieving recovery yields of 96.23% for CSNP A and 96.07% for CSNP B. The initial weight of native cassava starch was 20 g, representing a 100% starting yield. The effectiveness of the selected hydrolysis conditions is demonstrated by the slight yield reduction following hydrolysis, which suggests little degradation. These yields are notably higher than those reported by prior studies, including Hamad and Hu [16], Corre and Angellier-Coussy [17], and Shahrodin et al. [8], which employed stronger mineral acids, such as HCl and H_2_SO_4_, resulting in substantially lower yields due to excessive starch degradation.

The use of acetic acid for the hydrolysis of cassava starch in this study presents a new approach compared to the more commonly used inorganic acids like HCl and H_2_SO_4_ [13]. Acetic acid’s milder, organic nature protects the structural integrity of starch granules better than stronger acids, so using it for hydrolysis introduces a more controlled process. Acetic acid selectively promotes hydrolysis to maintain a higher yield, without significantly disrupting the glycosidic bonds within the crystalline regions. This approach aligns with recent trends favoring weaker organic acids in nanoparticle synthesis due to their biodegradability, lower corrosivity, and minimal environmental impact compared to mineral acids [13,14,18].

The high recovery yield was further enhanced by the application of ultrasonication in conjunction with acid hydrolysis. Agi et al. [15] and Dinari and Mallakpour [19] confirmed that ultrasonication can improve particle size reduction without causing significant mass loss. Their results prove that ultrasonic waves foster the cleavage of glycosidic bonds in the amorphous portions of starch, allowing the formation of nanoparticles without affecting the crystalline structure. In comparison to traditional mechanical mixing techniques, which result in lower recovery rates because of extended exposure and uneven treatment of the starch granules, this procedure was shown to be both efficient and time-effective.

The preservation of crystalline structure post-hydrolysis is vital for CSNP applications, particularly in enhanced oil recovery, where structural stability under thermal and mechanical stress is essential. Strong acids, such as HCl and H_2_SO_4_, which have higher dissociation constants, cause excessive degradation that compromises crystalline regions, leading to lower yields and less stable nanoparticles [16,20,21,22]. The findings herein suggest that the optimized acid hydrolysis protocol with acetic acid, enhanced by ultrasonication, provides a promising pathway for high-yield, structurally intact starch nanoparticles suitable for high-performance industrial applications.

#### 3.1.2. Morphological Properties and Particle Size Distribution

**Figure 2:**
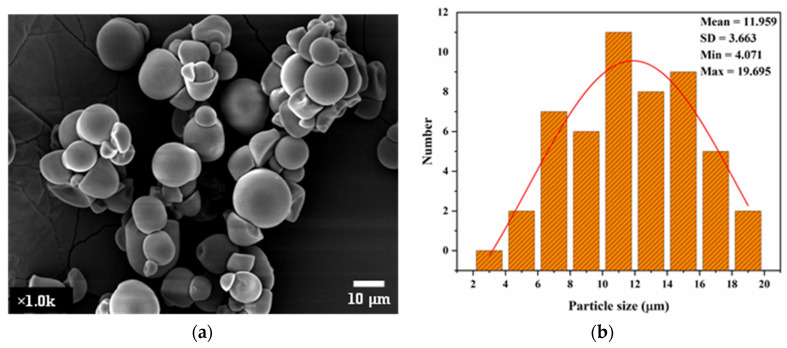
(**a**) SEM image of native cassava starch (CS), illustrating irregularly shaped granules with smooth surfaces and diameters ranging from ~4 to 19 µm. (**b**) Representative particle size distribution (PSD) obtained from microscopy analysis of native CS granules.

**Figure 3:**
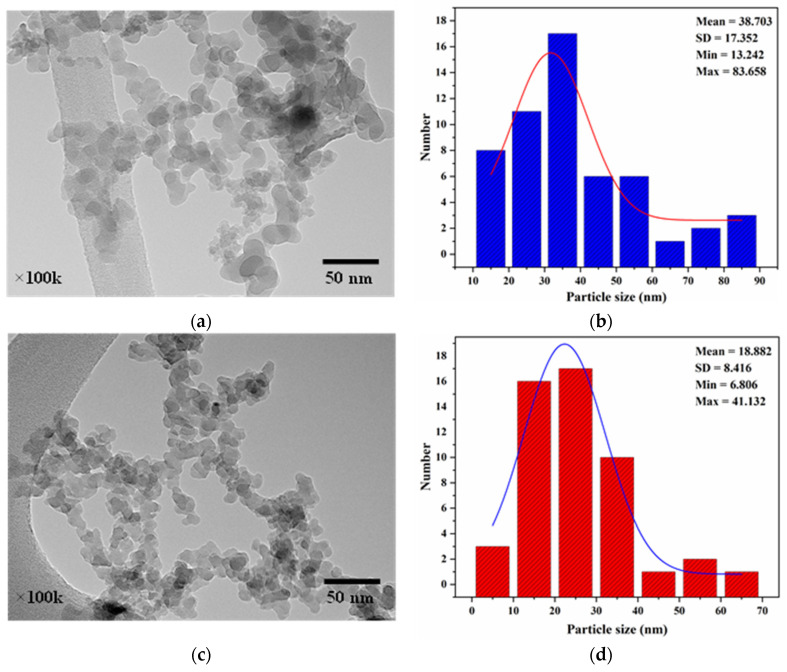
TEM images of (**a**) CSNP A and (**c**) CSNP B, along with corresponding particle size distribution histograms for (**b**) CSNP A and (**d**) CSNP B. CSNP A shows particles predominantly in the 13–84 nm range, while CSNP B exhibits smaller nanoparticles averaging about 19 nm in diameter.

Figure 2 depicts the SEM image and particle size distribution of native CS, revealing irregularly shaped granules with smooth surfaces ranging from 4 to 19 μm in diameter, consistent with findings reported by Rahaman et al. [23], Xie et al. [24], and Zhu [25]. This microstructure was significantly altered post-hydrolysis, as observed in the TEM images of CSNPs (Figure 3), where CSNP A and CSNP B were seen as aggregates due to interparticle hydrogen bonding of surface hydroxyl groups. The CSNPs exhibited spherical and elliptical nanostructures, aligning with prior reports on tapioca starch nanoparticles by Saeng-On and Aht-Ong [26], and Hedayati et al. [27].

**Figure 4:**
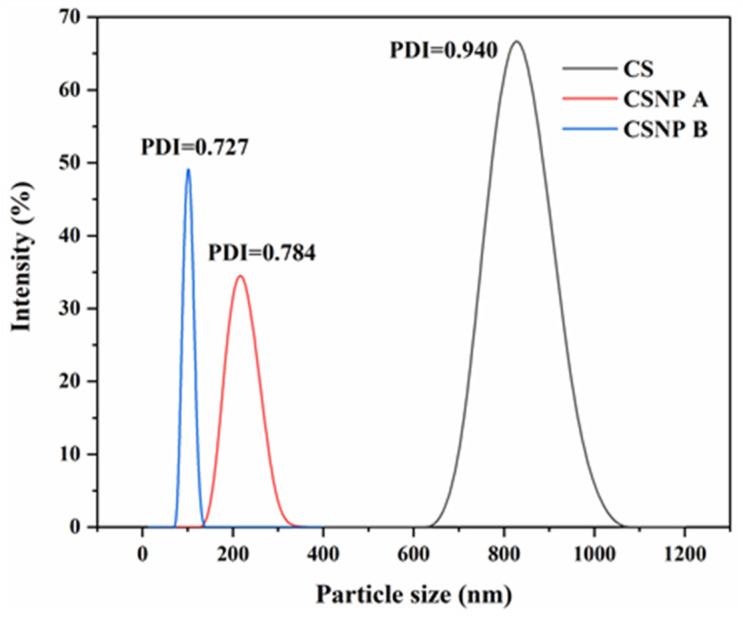
DLS particle size distribution of CS, CSNP A, and CSNP B.

A marked reduction in particle size was achieved through optimized hydrolysis. TEM analysis demonstrated that CSNP B, synthesized under a 3.49 M acetic acid concentration, a temperature of 36.6 °C, and a reaction time of 7 days, yielded particles between 6 and 41 nm as shown in Figure 3d. In comparison, CSNP A, obtained with a milder acid concentration of 2.34 M at 30 °C and a shorter reaction duration of 3 days, exhibited a particle size distribution from 13 to 83 nm (Figure 3b). The smaller particle size of CSNP B reflects the enhanced cavitation effect facilitated by ultrasonication, which is more pronounced at higher temperatures, causing microbubbles to collapse rapidly and effectively fragment starch granules [28,29]. During ultrasonication, the CSNP B suspension was at a slightly higher temperature than the CSNP A suspension. Therefore, cavitation bubbles formed more easily in the CSNP B suspension compared to the CSNP A suspension. This was because a higher temperature facilitates cavitation forces by increasing the vapor pressure of the solvent [30]. As a result, the CSNP B particles were much smaller than the CSNP A particles, which was also confirmed using the dynamic light scattering (DLS) method, as shown in Figure 4.

Figure 4 shows the DLS particle size distribution of CS, CSNP A, and CSNP B. The starch particles in the dry state could be visually compared using SEM and TEM images. To obtain a statistical result, the particle size intensity distribution of both the native CS and nanoparticles was determined using the light scattering method. DLS was used to further characterize the particle size of starch in an aqueous suspension (wet state). This method determined the hydrodynamic diameter by considering the Brownian motion of the nanoparticles in the suspension [31]. DLS results indicated that native CS had a mean diameter of 825 nm, which reduced to 215.5 nm for CSNP A and 99.8 nm for CSNP B, as summarized in Table 1. The discrepancy between the DLS and TEM measurements is attributed to the swelling and formation of an electrical double layer around the CSNPs in aqueous suspension, affecting hydrodynamic diameter readings in the wet state compared to dry-state TEM values. The polydispersity index (PDI) values of 0.784 for CSNP A and 0.727 for CSNP B (Table 1) indicate a relatively broad particle size distribution (PDI > 0.2), which is typical for acid-hydrolyzed starch nanoparticles due to heterogeneous granule fragmentation during ultrasonication and hydrolysis [27]. While a narrower PDI is preferable for uniform injectivity and flow in porous media, the observed shear-thickening behavior and high viscosity of CSNPs (Section 3.1.3) may compensate for this variability by enhancing mobility control, particularly in low-permeability reservoirs where smaller particles dominate. Future optimization could focus on reducing PDI through refined hydrolysis conditions to further improve EOR performance [32,33].

The differences in the particle sizes between CSNP A and CSNP B have implications for their application in enhanced oil recovery (EOR). CSNP B with a smaller particle size and larger surface area has more interaction with reservoir fluids, higher viscosity, and better mobility ratio for polymer flooding [34]. Therefore, it is more suitable for low-permeability reservoirs where deep penetration and more oil displacement are required [35,36]. However, the smaller particle size of CSNP B may cause pore blocking in high-permeability reservoirs, impede fluid flow, and reduce recovery efficiency.

Conversely, CSNP A with a particle size of 215.5 nm is more suitable for high-permeability reservoirs where pore-blocking risk is minimal [37,38]. Also, CSNP A requires less energy and a shorter reaction time, so it is a cost-effective option for large-scale EOR applications, although it has a slightly lower viscosity than CSNP B. These findings underscore the importance of selecting the appropriate CSNP formulation tailored to specific reservoir conditions, highlighting the trade-offs between particle size and performance for optimal EOR effectiveness.

#### 3.1.3. Rheological Properties

**Figure 5:**
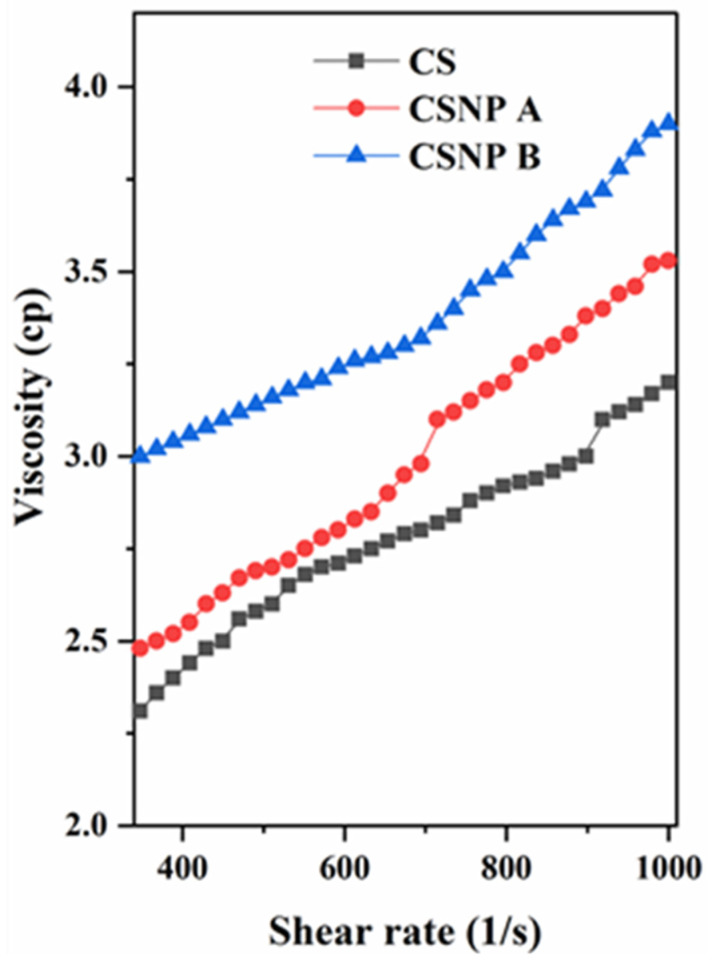
Viscosity of starch suspension at a concentration of 2000 ppm.

Figure 5 illustrates the viscosity profiles of native cassava starch (CS), CSNP A, and CSNP B suspensions across varying shear rates. Notably, the CSNP A and CSNP B suspensions exhibit increased apparent viscosity following acid hydrolysis, with CSNP B demonstrating the highest viscosity. This effect is attributed to the substantial surface area of the nanoparticles, which fosters stronger inter-particle interactions and, consequently, a higher viscosity for nano-sized starch suspensions compared to the micron-sized native starch suspension. Shear-thickening behavior was observed across all samples, indicated by a rise in shear viscosity as the shear rate increased from 350 to 1000 s^−1^ [39]. This phenomenon was particularly pronounced as particle size decreased, leading to more significant shear-thickening in CSNP suspensions. This observation aligns with findings from previous studies [40], which noted similar behavior in silica nanoparticles where smaller particles of 15 nm size exhibited higher shear-thickening effect than larger particles (2 μm) due to enhanced susceptibility to shear fields and stronger inter-particle interactions even at lower particle concentrations.

Moreover, reduced exposure of interaction sites on the polymer chains likely promotes aggregation and alignment under higher shear rates, increasing viscosity as the molecular alignment stabilizes [41]. The unique amylopectin structure within the starch may contribute to shear-thickening, as it allows aggregation under shear stress, enhancing the rheological response [42]. Additionally, negative ion charges and hydrophobic interactions also facilitate further hydrogen bonding and electrostatic interactions, collectively reinforcing the observed shear-thickening effect.

**Figure 6:**
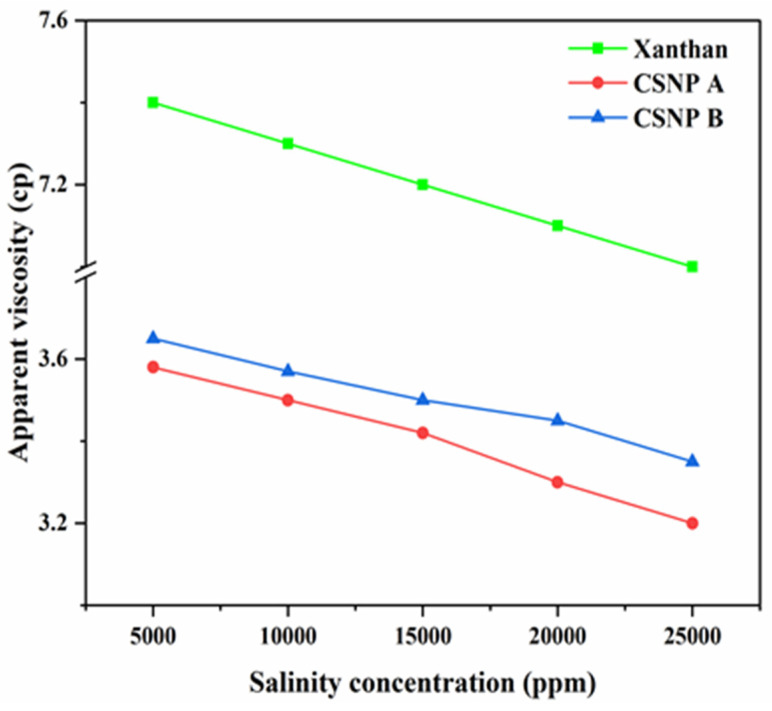
The apparent viscosity of starch suspension at different salinity concentrations at a specific shear rate of 1000 s^−1^.

Moving forward, the effects of salinity on the apparent viscosity of CSNP A, CSNP B, and xanthan gum suspensions are illustrated in Figure 6. Tested across salinity levels from 5000 to 25,000 ppm (shear rate: 1000 s^−1^), all suspensions exhibited a decrease in apparent viscosity with increasing salinity. This reduction is attributed to the monovalent cations (Na^+^) providing a charge-screening effect, which diminishes segmental repulsion and reduces the hydrodynamic volume, resulting in a lower viscosity [43]. Significantly, NaCl addition did not change the shear-thickening behavior of the starch suspensions, and the viscosity continued to increase with the shear rate, consistent with prior findings [44].

Shear-thickening fluids were historically unfavorable in industrial processes due to their potential to obstruct fluid flow in narrow channels. However, recent studies highlight the utility of shear-thickening materials for applications requiring impact protection and shock absorption due to their increased viscosity under stress. In the context of enhanced oil recovery (EOR), this property is particularly advantageous as it mitigates viscosity loss in polymers, improving mobility control and reducing polymer dosage requirements [45]. This behavior supports the practical viability of CSNP suspensions as functional agents in EOR applications, where maintaining and controlling viscosity is essential for optimal oil displacement.

#### 3.1.4. Thermal Properties

**Figure 7:**
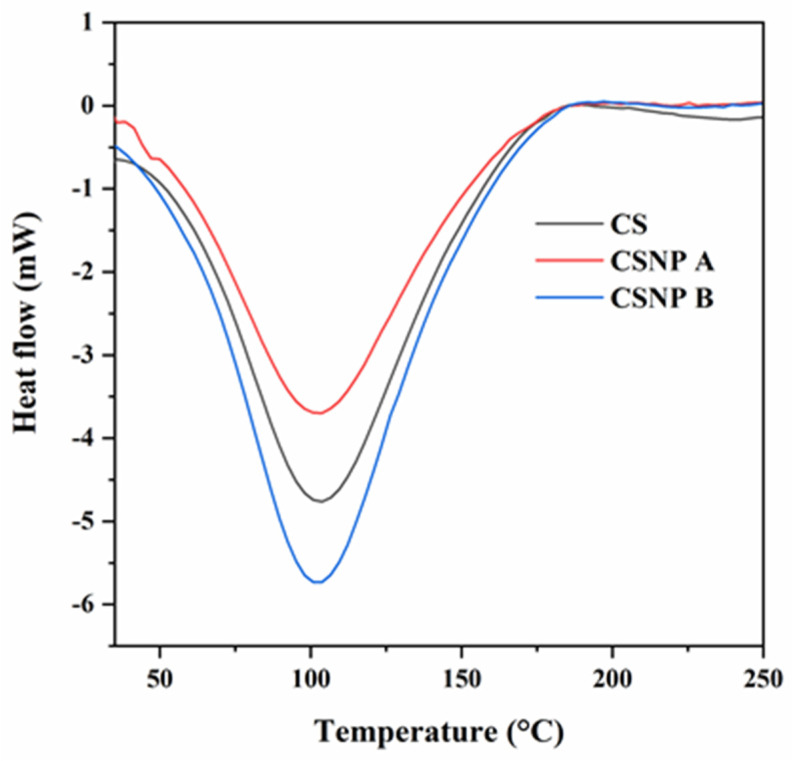
DSC thermograms of CS, CSNP A, and CSNP B.

The thermal properties of native cassava starch (CS), CSNP A, and CSNP B were investigated using differential scanning calorimetry (DSC) and thermogravimetric analysis (TGA). DSC provides insights into the enthalpy changes (ΔH) associated with starch gelatinization, a process where heat transforms starch from a crystalline to a gel state [46]. This transition temperature, referred to as the gelatinization temperature, is a critical parameter in starch characterization. The DSC thermograms (Figure 7) display a single endothermic peak for each sample, with ΔH values of −282.62 J/g, −247.02 J/g, and −343.37 J/g for CS, CSNP A, and CSNP B, respectively. CSNP B, which underwent the longest hydrolysis period (7 days), showed the broadest endothermic peak. This observation aligns with findings by Aparicio-Saguilan et al. [47], who reported that broader thermogram peaks indicate increased heterogeneity in crystalline structures. Table 2 summarizes the onset (*T_o_*), peak (*T_p_*), and conclusion (*T_c_*) temperatures, as well as ΔH values for each sample.

The DSC data reveal distinct thermal behaviors for CSNP A and CSNP B. At a shorter hydrolysis time (3 days), CSNP A exhibited increased *T_o_* and *T_c_* values but a lower ΔH compared to CSNP B. The higher *T_o_* and *T_c_* for CSNP A suggest a more organized crystalline region, primarily amylopectin, which requires elevated temperatures to undergo gelatinization [15]. In contrast, the lower T_o_ and broader endothermic peak observed in CSNP B suggest enhanced internal plasticization due to increased structural heterogeneity from prolonged hydrolysis [48]. Furthermore, the higher ΔH in CSNP B reflects increased crystallinity, indicating that greater energy is required to disrupt its organized starch matrix, likely due to the higher amylopectin content [14,49].

**Figure 8:**
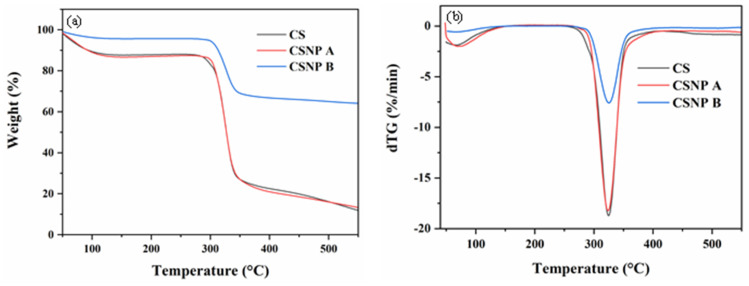
TGA and DTG curves of CS, CSNP A, and CSNP B: (**a**) Weight loss (%) vs. temperature; (**b**) Derivative weight loss (%/min) vs. temperature.

TGA and differential TG (dTG) analyses were used to evaluate the thermal stability and decomposition behavior of CS and the CSNPs. Figure 8 displays the TG and dTG curves, highlighting two primary weight-loss events for each sample. The first, occurring between 48.6 °C and 53.8 °C, corresponds to water loss due to dehydration [50,51,52,53], as shown in Table 3. CS and CSNP A showed higher moisture loss than CSNP B, indicating that these samples retained more bound and adsorbed water, likely due to their amorphous, less compact structures. These findings are in agreement with Garcia et al. [53], who attributed higher moisture content to increased hydroxyl groups that form hydrogen bonds with water molecules. In contrast, CSNP B exhibited minimal water loss (4.30%), suggesting a denser, more organized structure, likely reinforced by acetic acid esterification that limits hydroxyl exposure and reduces water retention.

The second weight-loss stage, between 278 °C and 287 °C, represents the decomposition of organic components, including glycerol release and depolymerization of carbon chains [53]. Both CS and CSNP A displayed similar degradation patterns, with approximately 60% weight loss. CSNP B, however, showed slightly higher decomposition onset (287 °C) and significantly lower weight loss (26.95%), highlighting its superior thermal stability. The increased residual content in CSNP B (63.5%) compared to CS (8.2%) and CSNP A (10.44%) suggests a highly organized structure, likely due to closer molecular packing from acetic acid hydrolysis, resulting in a denser starch framework and increased carbon residue after thermal degradation. Thus, the enhanced thermal stability and crystallinity observed in CSNP B, attributable to prolonged acid hydrolysis, make it an ideal candidate for applications requiring robust, thermally stable materials. The distinct thermal properties of CSNP A and CSNP B underscore the influence of hydrolysis duration on starch nanoparticle structure and provide insights for selecting optimal processing conditions based on specific application requirements.

#### 3.1.5. Chemical Structure

**Figure 9:**
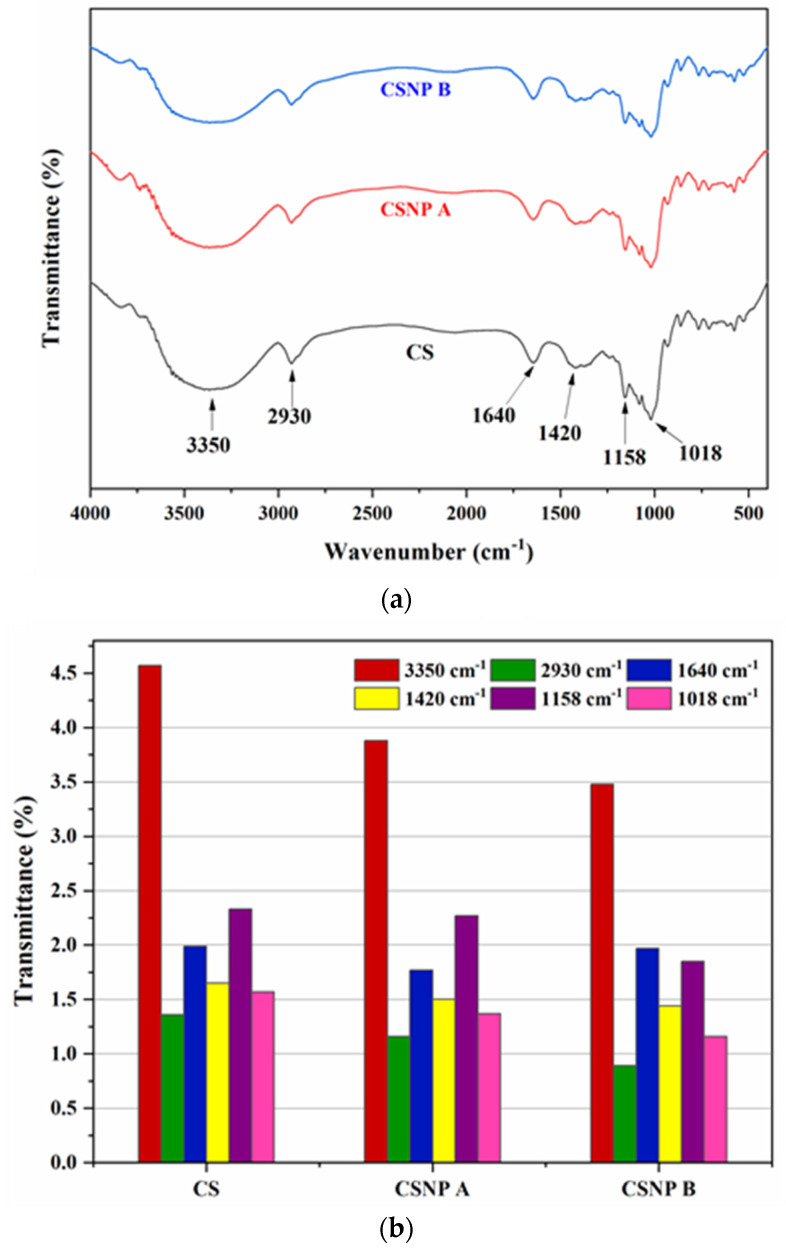
(**a**) FTIR spectra and (**b**) peak intensity for each functional group wavenumber of CS, CSNP A, and CSNP B.

The chemical structure of native cassava starch (CS) and cassava starch nanoparticles (CSNPs) post-acid hydrolysis was investigated using Fourier transform infrared (FTIR) spectroscopy. The FTIR spectra (Figure 9a) of CS, CSNP A, and CSNP B reveal no significant shifts in peak positions across the 400–4000 cm^−1^ range, indicating that the fundamental structural framework of starch remained intact. However, a noticeable reduction in peak intensity was observed after hydrolysis; this could be due to increased acid concentration, which decreases the amorphous regions, leading to lower absorbance intensities as can be seen in Figure 9b [54]. The hydrolyzed amorphous region during acid hydrolysis causes a reduction in the number of wavelengths absorbed by the samples, resulting in a decreased band peak intensity.

All samples exhibited a broad O-H stretching band at 3350 cm^−1^, associated with amylose and amylopectin units. This band was attenuated after hydrolysis, suggesting a reduction in bound water content due to structural reorganization during ultrasonication. Peaks observed at 2930 cm^−1^ and 1420 cm^−1^ correspond to C-H stretching, with their reduced intensity attributed to the ultrasonic treatment, as supported by Ahmed and Cui [55,56]. Meanwhile, the intense and sharp peak at 1640 cm^−1^, attributed to C=O stretching from ester groups, and a peak at 1158 cm^−1^, indicating C-O and C-O-C stretching within glycosidic linkages, were also present [12,23]. The region between 950 and 1068 cm^−1^, with a prominent peak at 1018 cm^−1^ and shoulders at 1047 cm^−1^ and 995 cm^−1^, aligns with the crystalline and amorphous regions of the starch structure [57].

To quantify crystallinity, the transmittance ratios R1047/1018 and R1018/995 were calculated, representing the crystalline and amorphous areas, respectively (see Table 4). Post-hydrolysis, CSNP B showed an increase in R1047/1018 and a decrease in R1018/995, indicating enhanced crystallinity [46]. These results align with those of Ahmed et al. [55], who reported similar changes in starch structure following size reduction. XRD analysis further confirmed the increase in relative crystallinity in CSNP B, highlighting a significant shift from the amorphous to crystalline state with prolonged hydrolysis.

#### 3.1.6. Crystalline Structure

**Figure 10:**
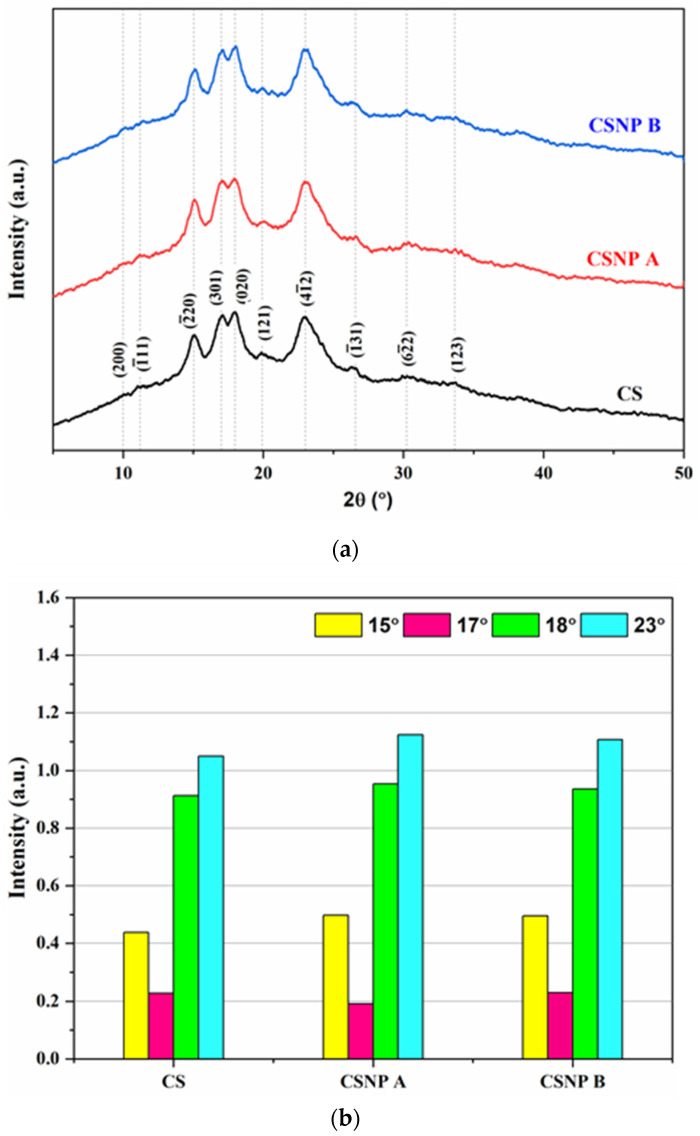
(**a**) XRD patterns, and (**b**) XRD peak intensities of native CS, CSNP A, and CSNP B.

X-ray diffraction (XRD) analysis was conducted to assess the crystallinity of CS, CSNP A, and CSNP B. The XRD patterns (Figure 10a) reveal A-type crystallinity across all samples, with characteristic peaks at 15° (−220), 23° (4−12), and a double peak at 17° (301) and 18° (020), consistent with prior studies [12,58]. The packing of double helices by amylopectin chains is more compact in the A-type crystalline structure than in the B-type diffraction pattern, while the C-type structure is a hybrid of the A and B patterns [27].

As shown in Table 5, the relative crystallinity (RC) increased from 12.8% in native CS to 13.1% and 14.5% in CSNP A and CSNP B, respectively. The extended hydrolysis duration and elevated acid concentration used for CSNP B promoted crystallization by selectively hydrolyzing amorphous regions, as noted in prior research on starch crystallization [59]. The increased intensity of diffraction peaks in CSNP A and CSNP B, compared to CS, further supports the higher crystallinity in the CSNPs, as shown in Figure 10b. This was because the amorphous area was more easily hydrolyzed than the crystalline area, resulting in an increase in the crystalline area with a loss in the amorphous portion after acid hydrolysis [26]. Polymer crystallinity has been such an essential feature in EOR applications as it is able to permit a more extensive enhancement of barrier properties to improve the stability of the polymers in the reservoir [60]. Therefore, it can prevent polymer degradation and eventually enhance the effectiveness of polymer flooding.

### 3.2. Modelling and Analysis

#### 3.2.1. Response Surface Methodology (RSM) and Experimental Design

The impact of three key independent variables—acid concentration (ϰ_1_), reaction temperature (ϰ_2_), and hydrolysis time (ϰ_3_)—on the physicochemical properties (yield, particle size, and viscosity) of CSNPs was investigated using response surface methodology (RSM). In this study, a central composite rotatable design (CCRD) was adopted, which is particularly well-suited for optimization purposes due to its ability to handle non-linear relationships between multiple variables. A total of 20 experimental runs were generated from the CCRD, comprising 8 factorial points, 6 axial points, and 6 center points, ensuring replication and robustness of the design. The design matrix and corresponding experimental responses, including yield, particle size, and viscosity, are presented in Table 5. Design-Expert software (version 11) was employed to model the process and evaluate the functional relationships between the independent variables and responses.

The experimental data were fitted to a quadratic polynomial model using the following equation:

(3) $$Y=\beta_{0}+\sum_{i=1}^{k}\beta_{i}\varkappa_{i}+\sum_{i=1}^{k}\beta_{ii}\varkappa_{i}^{2}+\sum \sum_{i<j}^{k}\beta_{ij}\varkappa_{i}\varkappa_{j}+\in$$

where *Y* is the predicted response, *β_0_* is the regression coefficient for the first-degree terms, *β_ii_* is the coefficient for the pure quadratic terms, *β_ij_* is the coefficient for the cross-product terms, and ∈ is the random error term. This model was used to predict the behavior of the responses based on the independent variables. To provide a comprehensive representation of the relationships, the specific quadratic models for yield, particle size, and viscosity, expressed in terms of coded factors, were derived as follows:

Yield = 93.95 − 5.48ϰ_1_ − 17.10ϰ_2_ − 4.56ϰ_3_ + 1.16ϰ_1_^2^ − 12.04ϰ_2_^2^ + 0.26ϰ_3_^2^ − 7.36ϰ_1_ϰ_2_ − 0.43ϰ_1_ϰ_3_ − 7.27ϰ_2_ϰ_3_ (4)

Particle size = 108.84 − 35.65ϰ_1 −_ 28.51ϰ_2 −_ 20.46ϰ_3_ + 17.81ϰ_1_^2^ + 1.11ϰ_2_^2^ + 24.33ϰ_3_^2^ + 22.44ϰ_1_ϰ_2_ − 8.29ϰ_1_ϰ_3_ − 28.31ϰ_2_ϰ_3_ (5)

Viscosity = 3.31 + 0.25ϰ_1_ + 0.017ϰ_2_ + 0.091ϰ_3_ − 0.085ϰ_1_^2^ − 0.19ϰ_2_^2^ + 0.13ϰ_3_^2^ + 0.062ϰ_1_ϰ_2_ − 0.16ϰ_1_ϰ_3_ − 0.063ϰ_2_ϰ_3_ (6)

The coefficients for each term, determined using least squares regression, are summarized in Table 6. These equations align with the significant terms identified in the subsequent Analysis of Variance (ANOVA) providing a clear pathway to predict and optimize responses based on the coded factors. The ANOVA analysis was conducted to evaluate the statistical significance of the model [61]. Tables S3–S5 in the Supplementary Materials present the ANOVA results for yield, particle size, and viscosity, respectively. The model’s F-values of 12.69 (yield), 4.31 (particle size), and 3.96 (viscosity) indicate significant relationships between the independent variables and the responses, with *p*-values less than 0.05 indicating statistical significance. These F-values were compared against the tabulated F-values at a significance level of α = 0.05. In each case, the calculated F-values exceeded the critical F-value, confirming the robustness of the model in predicting the responses [62].

#### 3.2.2. Regression Analysis and Model Adequacy

Based on Table S6 in the Supplementary Materials, the Pred. R^2^ of 0.4714 in the response yield was not as close to the Adj. R^2^ of 0.8470 as might typically be expected. However, a negative Pred. R^2^ in the particle size and viscosity responses suggested that the overall mean was a better predictor of both responses than the current models. In addition, the adequate precision that measured the signal-to-noise ratio showed a desirable value of greater than 4. The adequate precision values obtained in the yield, particle size, and viscosity responses of the model were 12.941, 7.422, and 6.775, respectively, indicating an adequate signal; hence this model could be used to optimize the design.

The R^2^ values for yield, particle size, and viscosity were 0.9195, 0.7952, and 0.7807, respectively. This indicates that the model explained 91.95%, 79.52%, and 78.07% of the variability in the corresponding response data. To validate the regression model, the predicted values were computed using the generalized polynomial regression equation, and these values were plotted against the actual experimental data [63]. As shown in Figure S2a–c in the Supplementary Materials, there was strong agreement between the predicted and actual values, with R^2^ values of 0.9219 (yield), 0.7952 (particle size), and 0.7808 (viscosity). This close match between the predicted and observed data underscores the robustness and applicability of the proposed regression model for analyzing the effects of acid hydrolysis conditions on the physicochemical properties of CSNPs. Thus, confirming the significance of the model generated by ANOVA.

#### 3.2.3. Interaction Effects and Response Surface Plots

Figures S3–S5 given in the Supplementary Materials, illustrate the three-dimensional response surface plots and corresponding contour plots used to further analyze the interaction effects between the variables. For each of the three responses, these plots show notable interactions between the variables. In Figure S3, for example, yield increased with temperature until it reached a critical point (45 °C), at which point it began to decline, most likely as a result of starch gelatinization at higher temperatures [64]. Similarly, Figure S4 shows that particle size decreases as acid concentration increases, which can be attributed to increased hydrolysis of glycosidic bonds in the starch structure [20]. However, no further reduction in particle size was observed at temperatures above 50 °C, likely due to the starch transitioning to a gel condition.

Figure S5 shows that viscosity increases with acid concentration and temperature peaks at 43 °C and then decreases at higher temperatures. The increase in viscosity is due to the decrease in particle size which enhances the surface charge of the nanoparticles and leads to electrostatic repulsion and increased suspension viscosity. Ultrasonication also contributed to this effect by creating a more organized structure, increasing internal resistance and thus viscosity [21].

Thus, the RSM model provided insights into the effects of acid concentration, temperature, and hydrolysis time on the physicochemical properties of CSNPs. The statistical analysis proved the significance of the model, and the optimization results provided the optimal conditions to produce CSNPs with high yield, small particle size, and desirable viscosity. These results demonstrate that the optimized process can produce CSNPs for EOR applications, which is a sustainable and efficient alternative to conventional polymers. The strong correlation between predicted and experimental values validates the use of the model for further optimization and scale-up.

### 3.3. Optimization

#### Optimization Scenarios A and B

To predict the optimal conditions for acetic acid hydrolysis and produce a maximum yield of CSNP with the smallest particle size in the shortest amount of time, multivariable models derived from the statistical experimental design were used. With acid concentration (ϰ_1_), reaction temperature (ϰ_2_), and reaction time (ϰ_3_) as the main variables, Table 7 lists the optimization criteria for two scenarios. The objective of the first scenario, termed Optimization A (labeled CSNP A), was to minimize particle size, maximize yield and viscosity, and minimize ϰ_1_, ϰ_2_, and ϰ_3_. The goal of this optimization was to create a CSNP that was both economical and had good performance metrics. For the second scenario, Optimization B, the variables were allowed to float and achieve the same response objectives—maximum yield and viscosity with the smallest particle size. The nanoparticles produced in this scenario were labeled CSNP B to develop the best CSNP possible.

Table 8 presents the predicted optimal conditions and corresponding measured values for both Optimization A and B, illustrating the robustness of the response surface methodology (RSM) approach within a 5% error margin. Under Optimization A (2.34 M acid, 30 °C, 3 days), the CSNPs exhibited a 96.23% yield, a 206.77 nm particle size, and a 3.53 cP viscosity. In contrast, Optimization B (3.49 M acid, 36.56 °C, 7 days) produced CSNPs with a 96.07% yield, a 99.4 nm particle size, and a 3.65 cP viscosity. These results confirm that the optimized process parameters reliably achieve the target outcomes for yield, particle size, and viscosity, thereby underscoring the effectiveness of the RSM optimization strategy.

## 4. Discussion

The present study successfully optimized the synthesis of cassava starch nanoparticles (CSNPs) using ultrasonic-assisted acetic acid hydrolysis, achieving high yields and desirable physicochemical properties tailored for enhanced oil recovery (EOR) applications. The optimization process, facilitated by response surface methodology (RSM), revealed that varying the acid concentration, temperature, and hydrolysis time significantly influenced the yield, particle size, and viscosity of the resulting CSNPs. Notably, the two optimized samples, CSNP A and CSNP B, demonstrated distinct characteristics that underscore the efficacy of the chosen synthesis parameters.

One of the most compelling outcomes of this study is the remarkably high recovery yields of CSNPs exceeding 96%, which contrasts with yields reported in previous studies employing strong inorganic acids such as hydrochloric acid (HCl) and sulfuric acid (H_2_SO_4_) [8,16,17]. The improved yield can be attributed to the selective hydrolysis achieved through the use of acetic acid, a milder organic acid that preferentially targets the amorphous regions of cassava starch while preserving the integrity of the crystalline domains. This selective degradation minimizes the excessive breakdown of glycosidic bonds within the crystalline regions, thereby maintaining the structural robustness of the nanoparticles. Additionally, the incorporation of ultrasonication plays a pivotal role by enhancing mass transfer and promoting the efficient cleavage of starch granules into nanoparticles without significant mass loss, as reported by Agi et al. [15] and Dinari and Mallakpour [19]. The combined effect of acetic acid concentration and ultrasonication maximized the yield and assisted in producing uniformly sized nanoparticles, which is critical for their performance in EOR applications. From an economic perspective, ultrasonication’s viability for large-scale starch hydrolysis is promising due to its ability to enhance reaction efficiency, reduce processing times, and achieve high yields (>96%) using a milder, less corrosive reagent like acetic acid compared to traditional strong-acid methods [8,15]. While energy input and equipment costs are considerations, industrial ultrasonication systems are increasingly scalable, as demonstrated in food and polymer processing [30], suggesting potential cost-effectiveness. A detailed cost-benefit analysis is planned for future scale-up studies to confirm its economic feasibility.

Morphological analysis through scanning electron microscopy (SEM) and transmission electron microscopy (TEM) revealed that the optimized CSNPs exhibited reduced particle sizes compared to native cassava starch. CSNP B, synthesized under more intensive hydrolysis conditions, displayed nanoparticles ranging from 6 to 41 nm, which is smaller than the 13 to 83 nm range observed for CSNP A. This reduction in particle size is advantageous for EOR as smaller nanoparticles offer a larger surface area, enhancing their interaction with reservoir fluids and improving the mobility ratio during polymer flooding. The smaller size of CSNP B facilitates deeper penetration into low-permeability reservoirs, thereby increasing oil displacement efficiency. However, it is essential to balance particle size to prevent pore-blocking in high-permeability reservoirs, a consideration that underscores the practical applicability of both CSNP A and CSNP B in different reservoir conditions.

Rheological assessments demonstrated that both CSNP A and CSNP B exhibited shear-thickening behavior, with CSNP B showing a higher viscosity compared to CSNP A and native cassava starch. The shear-thickening property is particularly beneficial for EOR as it ensures that the polymer solution maintains its viscosity under high shear rates encountered during injection, thereby improving mobility control and reducing the likelihood of viscous fingering [39,40]. The higher viscosity of CSNP B is attributed to its smaller particle size, which improves inter-particle interactions and electrostatic repulsion, leading to increased suspension viscosity. Moreover, the ultrasonication process contributes to a more organized nanoparticle structure, further augmenting internal resistance and viscosity [21]. These rheological improvements position CSNP B as a superior candidate for polymer flooding in EOR, particularly in scenarios demanding sustained viscosity for effective oil displacement.

Thermal analysis via differential scanning calorimetry (DSC) and thermogravimetric analysis (TGA) revealed that CSNP B possesses enhanced thermal stability and increased crystallinity compared to both native cassava starch and CSNP A. The DSC thermograms indicated that CSNP B required greater enthalpy changes for gelatinization, reflecting a more organized and crystalline starch matrix. This enhanced crystallinity, confirmed by X-ray diffraction (XRD) analysis, is crucial for maintaining structural integrity under the high-temperature conditions prevalent in many oil reservoirs [50]. The TGA results further demonstrated the thermal robustness of CSNP B, showing a higher decomposition onset temperature and significantly lower weight loss, which indicate a denser and more stable nanoparticle structure. These thermal properties ensure that CSNP B remains effective and stable during the injection process and prolonged exposure to reservoir conditions.

Fourier transform infrared (FTIR) spectroscopic analysis confirmed that the fundamental chemical structure of cassava starch remained intact post-hydrolysis, with no significant shifts in peak positions. However, a reduction in peak intensity suggested a decrease in bound water content and a more compact structure due to selective hydrolysis. The increased relative crystallinity observed in CSNP B, as evidenced by higher R1047/1018 and lower R1018/995 ratios, aligns with the XRD findings and indicates a successful enhancement of the crystalline regions. These structural modifications are integral to resisting the harsh thermal, salinity, and mechanical stresses typically encountered during EOR.

The successful application of RSM optimization confirms that the interplay of acid concentration, temperature, and reaction time can be fine-tuned to yield desirable nanoparticle sizes, high recovery percentages, and robust rheological characteristics. This outcome not only validates the effectiveness of RSM in complex, multivariable systems but also provides a roadmap for industrial-scale production of CSNPs tailored to specific reservoir requirements. Given the reproducibility and accuracy of the optimized conditions, scaling up the current laboratory approach to pilot or commercial levels appears feasible. Moreover, the use of acetic acid, with its lower corrosiveness and environmental footprint, aligns with the industry’s move toward more sustainable chemical processes. The favorable rheological performance and enhanced thermal stability of CSNP B highlight its potential as an alternative or complementary agent to conventional EOR polymers, which often face degradation under high-temperature or high-salinity conditions. By retaining high viscosity and ensuring stable flow characteristics over the lifespan of the flooding process, CSNP B can help mitigate common issues such as polymer breakdown and rapid viscosity loss. Although field trials will be necessary to verify laboratory outcomes in real reservoir environments, the demonstrated synergy between green processing, high yields, and strong performance underlines the relevance of this approach in addressing global energy challenges.

### Preliminary Assessment of CSNPs for EOR Applications

The primary objective of synthesizing cassava starch nanoparticles (CSNPs) in this study was to develop a sustainable, high-performance material tailored for enhanced oil recovery (EOR) through polymer flooding. While direct oil recovery experiments, such as core flooding, were not conducted in this initial investigation due to its focus on synthesis optimization and characterization, the physicochemical properties of CSNP A and CSNP B provide a strong foundation for assessing their potential applicability in EOR.

CSNP B, with an average particle size of 99.4 nm (DLS), exhibits a high apparent viscosity and pronounced shear-thickening behavior (Figure 5), which are critical for improving the mobility ratio between oil and water phases during polymer flooding [1,39]. The smaller particle size enhances its ability to penetrate low-permeability reservoirs, increasing sweep efficiency and oil displacement, as smaller nanoparticles are known to improve fluid interaction and reduce pore-blocking risks in such conditions [34,35]. Conversely, CSNP A, with a larger particle size of 206.77 nm, may be better suited for high-permeability reservoirs where pore clogging is less of a concern, offering a cost-effective alternative due to its shorter synthesis time (3 days vs. 7 days for CSNP B) [37,38]. The shear-thickening property observed in both CSNPs ensures that viscosity is maintained or even increases under the high shear rates encountered during injection, mitigating the viscosity loss commonly observed in conventional EOR polymers like hydrolyzed polyacrylamide (HPAM) under similar conditions [2,45]. This behavior is particularly advantageous for maintaining mobility control and reducing polymer dosage requirements in EOR operations.

Thermal stability is another critical factor for EOR applications, given the elevated temperatures often encountered in oil reservoirs. CSNP B demonstrated superior thermal stability, with a decomposition onset temperature of 287 °C and a residual mass of 63.5% as shown in Table, compared to CSNP A (285.9 °C, 10.5% residual) and native CS (278 °C, 8.2% residual). This enhanced stability, coupled with increased crystallinity (14.5% RC, Table 4), suggests that CSNP B can withstand prolonged exposure to high-temperature and high-salinity environments without significant degradation, a common limitation of biopolymers like xanthan gum [2,60]. The ability of CSNPs to maintain structural integrity under such conditions supports their potential to outperform or complement synthetic polymers in harsh reservoir settings.

Furthermore, the high recovery yields (>96%) achieved for both CSNP variants highlight the efficiency of the ultrasonic-assisted acetic acid hydrolysis process, ensuring scalability and economic viability for industrial EOR applications. The use of a biodegradable, eco-friendly material like cassava starch also aligns with the industry’s shift toward sustainable practices, offering an environmentally responsible alternative to inorganic nanoparticles or synthetic polymers [4,5].

While these properties strongly indicate that CSNP B, in particular, is a promising candidate for EOR polymer flooding, we acknowledge that laboratory-scale core flooding experiments and field trials are essential to quantify oil recovery efficiency and validate injectivity under realistic reservoir conditions. Such studies will assess parameters like residual oil saturation, displacement efficiency, and long-term stability in porous media, providing conclusive evidence of CSNPs’ practical utility. These next steps are planned as part of our ongoing research to bridge the gap between laboratory characterization and field application, ensuring that the potential demonstrated here translates to tangible EOR performance.

## 5. Conclusions

This study successfully optimized the synthesis of cassava starch nanoparticles (CSNPs) using ultrasonic-assisted acetic acid hydrolysis, guided by response surface methodology (RSM), to produce two variants—CSNP A and CSNP B—with high recovery yields exceeding 96%, surpassing those of conventional inorganic acid methods. CSNP B, characterized by a smaller particle size (99.4 nm), exhibited superior rheological properties, including higher viscosity and pronounced shear-thickening behavior, alongside enhanced thermal stability (decomposition onset at 287 °C, 63.5% residual mass) and crystallinity (14.5% RC). These attributes position CSNP B as a promising candidate for enhanced oil recovery (EOR) polymer flooding, particularly in low-permeability, high-temperature, and high-salinity reservoirs, where sustained viscosity and structural integrity are paramount. CSNP A, with a larger particle size (206.77 nm) and shorter synthesis time, offers a cost-effective alternative for high-permeability reservoirs. FTIR and XRD analyses confirmed the nanoparticles’ increased crystallinity, while DSC and TGA underscored their thermal robustness, critical for EOR applications. A preliminary assessment based on these properties suggests that CSNPs, especially CSNP B, can enhance mobility control and oil displacement efficiency in EOR by maintaining viscosity under high shear rates and resisting degradation in harsh reservoir conditions. However, to fully validate their efficacy, future work must include laboratory core flooding experiments and field trials to quantify oil recovery performance under realistic conditions. Scaling up the synthesis process also remains a key step toward industrial applicability. This study lays a strong foundation for developing sustainable, high-performance biopolymer nanoparticles, offering a viable alternative to conventional EOR agents and contributing to greener oil recovery practices.

## Figures and Tables

**Table 1:** Mean particle size of native and nanoparticles determined by SEM/TEM and DLS at room temperature.

Sample	SEM/TEM			DLS	
	Diameter (nm)			Hydrodynamic Diameter (nm)	
	Min	Max	Mean ± SD	Mean	PDI
Native CS	4.1 × 10^3^	19.7 × 10^3^	12.0 × 10^3^ ± 3.7	825.0	0.940
CSNP A	13.2	83.7	38.7 ± 17.4	215.5	0.784
CSNP B	6.8	41.1	18.9 ± 8.4	99.8	0.727

**Table 2:** DSC parameters of CS, CSNP A, and CSNP B.

Sample	T_o_ (°C)	T_p_ (°C)	T_c_ (°C)	∆H (J/g)
CS	47	104	175	−282.6
CSNP A	50	103	183	−247.1
CSNP B	41	102	174	−343.4

T_o_: Onset temperature, T_p_: Peak temperature, T_c_: Conclusion temperature, ∆H: Enthalpy.

**Table 3:** Thermal degradation parameters of CS, CSNP A, and CSNP B.

Sample	T_o_ (°C)/Weight Loss (%)		Residual (%)
	1st Event	2nd Event	
CS	49/12.2	278/60.8	8.2
CSNP A	49/12.9	286/60.9	10.5
CSNP B	54/4.3	287/26.9	63.5

T_o_: Onset temperature.

**Table 4:** Relative Crystallinity and Transmittance Ratios for CS, CSNP A, and CSNP B.

Sample	RC (%)	R_1047/1018_	R_1018/995_
CS	12.8	1.14	0.85
CSNP A	13.1	1.13	0.86
CSNP B	14.5	1.18	0.82

**Table 5:** Design of the experiment and measured responses.

Run No.	Factor			Response		
	ϰ_1_ (M)	ϰ_2_ (°C)	ϰ_3_ (d)	Yield (%)	Size (nm)	Viscosity (cP)
1	3.60	50.00	7.00	36.5	85.9	3.6
2	3.60	50.00	3.00	68.8	131.6	3.6
3	2.20	30.00	7.00	97.5	316.1	3.4
4	2.90	40.00	5.00	89.0	97.8	3.5
5	2.20	30.00	3.00	98.9	215.4	2.5
6	3.60	30.00	7.00	96.9	130.8	3.4
7	3.60	30.00	3.00	93.2	147.9	3.6
8	2.20	50.00	7.00	73.4	96.8	2.9
9	2.20	50.00	3.00	97.1	194.0	2.7
10	2.90	40.00	1.64	98.1	225.1	3.5
11	1.72	40.00	5.00	99.0	189.0	2.8
12	2.90	40.00	5.00	98.4	90.1	3.6
13	2.90	40.00	5.00	90.1	84.3	3.0
14	2.90	23.18	5.00	97.2	120.0	2.6
15	2.90	40.00	8.36	93.0	94.2	3.7
16	2.90	56.82	5.00	24.2	67.9	2.8
17	4.08	40.00	5.00	97.1	93.4	3.2
18	2.90	40.00	5.00	99.5	130.6	3.4
19	2.90	40.00	5.00	97.1	137.9	3.1
20	2.90	40.00	5.00	89.5	118.5	3.3

**Table 6:** Coefficients of the final equations for each response.

Coefficient	Yield	Particle Size	Viscosity
Intercept	93.95	108.84	3.31
ϰ_1_	−5.48	−35.65	0.25
ϰ_2_	−17.1	−28.51	0.017
ϰ_3_	−4.56	−20.46	0.091
ϰ_1_^2^	1.16	17.81	−0.085
ϰ_2_^2^	−12.04	1.11	−0.19
ϰ_3_^2^	0.26	24.33	0.13
ϰ_1_ϰ_2_	−7.36	22.44	0.062
ϰ_1_ϰ_3_	−0.43	−8.29	−0.16
ϰ_2_ϰ_3_	−7.27	−28.31	−0.063

**Table 7:** Goals for Optimization A and Optimization B.

	Goal	Lower Limit	Upper Limit
**Optimization A**			
ϰ_1_ (M)	Minimize	2.2	3.6
ϰ_2_ (°C)	Minimize	30	50
ϰ_3_ (d)	Minimize	3	7
Yield (%)	Maximize	24.2	99.5
Size (nm)	Minimize	67.9	316.1
Viscosity (cP)	Maximize	2.5	3.7
**Optimization B**			
ϰ_1_ (M)	In Range	2.2	3.6
ϰ_2_ (°C)	In Range	30	50
ϰ_3_ (d)	In Range	3	7
Yield (%)	Maximize	24.2	99.5
Size (nm)	Minimize	67.9	316.1
Viscosity (cP)	Maximize	2.5	3.7

**Table 8:** The predicted and measured values for conditions in Optimizations A and B.

Optimization	Predicted Values	Measured Values	Error (%)
**Optimization A**			
Acid concentration (M)	2.34	2.34	–
Temperature (°C)	30	30	–
Time (d)	3	3	–
Yield (%)	95.16	96.23	1.12
Size (nm)	206.71	206.77	0.03
Viscosity (cP)	3.33	3.53	5.11
**Optimization B**			
Acid concentration (M)	3.49	3.49	–
Temperature (°C)	36.56	36.56	–
Time (d)	7	7	–
Yield (%)	94.58	96.07	1.58
Size (nm)	101.47	99.4	−2.04
Viscosity (cP)	3.51	3.65	3.99

## Data Availability

The original contributions presented in this study are included in the article. Further inquiries can be directed to the corresponding author.

## Supplementary Materials

The following supporting information can be downloaded at: https://www.mdpi.com/article/10.3390/polym17081071/s1. Figure S1. CCRD design for the optimization of three variables. (●) Points of factorial design, (○) axial points, and (□) central points. Table S1. Experimental range and coded levels for acid concentration, temperature, and time. Table S2. Central composite rotatable design (CCRD) matrix with trial number, run order, point type, and coded levels. Figure S2. Predicted vs. actual plots for model fitting: (a) yield, (b) particle size, and (c) viscosity. Figure S3. Response surface and contour plots showing the effect of acid concentration and temperature on yield at a fixed hydrolysis time. Figure S4. Response surface and contour plots showing the effect of acid concentration and temperature on particle size. Figure S5. Response surface and contour plots showing the effect of acid concentration and temperature on viscosity. Table S3. ANOVA table for the response surface quadratic model for yield. Table S4. ANOVA table for the response surface quadratic model for particle size. Table S5. ANOVA table for the response surface quadratic model for viscosity. Table S6. Regression coefficients and statistical parameters (R^2^, Adj. R^2^, Pred. R^2^, etc.) for yield, particle size, and viscosity.
