# Supplementary material for: Optimization and Characterization of Acetic Acid-Hydrolyzed Cassava Starch Nanoparticles for Enhanced Oil Recovery Applications
Source: Polymers (Basel). 2025 Apr 16;17(8):1071. doi: 10.3390/polym17081071 (PMC12030524; doi:10.3390/polym17081071)
Supplement: Supplementary file 1 [file polymers-17-01071-s001.zip › polymers-3532853-supplementary.pdf]

polymers

Supplementary Materials

Optimization and Characterization of Acetic Acid Hydrolyzed Cassava Starch Nanoparticles for Enhanced Oil Recovery Applications

Mohammed E. Ali Mohsin [1,*], A. F. A. Rahman [2], Zakiah Harun [2], Agus Arsad [2,*], Suleiman Mousa [1], Muhammad Abbas Ahmad Zaini [3], Mohammad Yousef Younes [1] and Mohammad Faseeulla Khan [4]

1 Department of Chemical Engineering, College of Engineering, King Faisal University, Al Ahsa 31982, Saudi Arabia; saamousa@kfu.edu.sa (S.M.); myounes@kfu.edu.sa (M.Y.Y.)
2 UTM-MPRC Institute for Oil and Gas, Faculty of Chemical and Energy Engineering, Universiti Teknologi Malaysia, 81310 Johor Bahru, Johor, Malaysia
3 Centre of Lipids Engineering & Applied Research, Ibnu-Sina Institute for Scientific & Industrial Research, Universiti Teknologi Malaysia, 81310 Johor Bahru, Johor, Malaysia
4 Department of Mechanical Engineering, College of Engineering, King Faisal University, Al-Ahsa 31982, Saudi Arabia
* Correspondence: maa.ali@kfu.edu.sa (M.E.A.M.); agus@utm.my (A.A.)

Figure S1. CCRD design for the optimization of three variables. (●) Points of factorial design, (○) axial points and (□) central points.

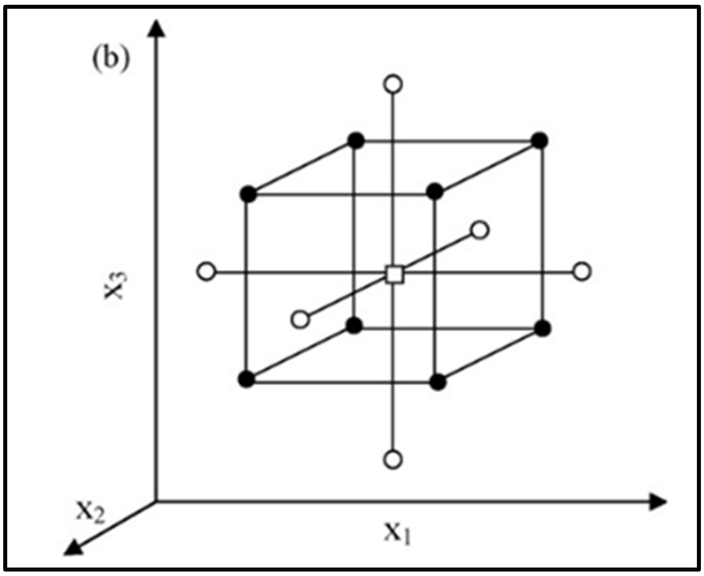

Table S1. Experimental range and coded level.

Independent variables	Symbol	Level				
		**-α**	**-1**	**0**	**1**	**+α**
**Acid concentration (M)**	$\varkappa_1$	1.72	2.20	2.90	3.60	4.08
**Temperature (°C)**	$\varkappa_2$	23.18	30	40	50	56.82
**Time (d)**	$\varkappa_3$	1.64	3	5	7	8.36

Table S2. Central composite rotatable design (CCRD) matrix.

Trial	Run no.	Points type	Coded levels		
			$\varkappa_1$	$\varkappa_2$	$\varkappa_3$
**1**	1	Factorial	1	1	1
**2**	2	Factorial	1	1	-1
**3**	3	Factorial	-1	-1	1
**4**	5	Factorial	-1	-1	-1
**5**	6	Factorial	1	-1	1
**6**	7	Factorial	1	-1	-1
**7**	8	Factorial	-1	1	1
**8**	9	Factorial	-1	1	-1
**9**	10	Axial	0	0	-α
**10**	11	Axial	-α	0	0
**11**	14	Axial	0	-α	0
**12**	15	Axial	0	0	α
**13**	16	Axial	0	α	0
**14**	17	Axial	α	0	0
**15**	4	Centre	0	0	0
**16**	12	Centre	0	0	0
**17**	13	Centre	0	0	0
**18**	18	Centre	0	0	0
**19**	19	Centre	0	0	0
**20**	20	Centre	0	0	0

Figure S2. The predicted and actual linear fitting of (a) yield, (b) particle size, and (c) viscosity**.**

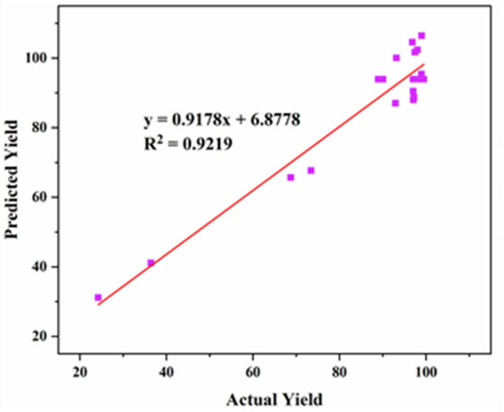

(a)

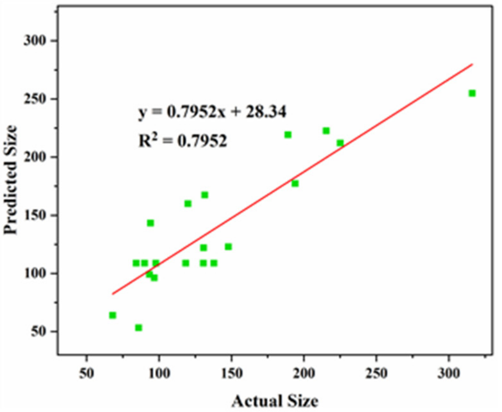

(b)

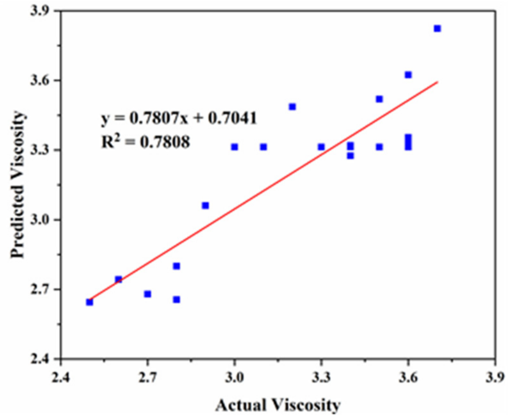

(c)

Figure S3. Response surface and contour plots showing the effect of acid concentration (x_1) and temperature (x_2) on yield at a fixed hydrolysis time.

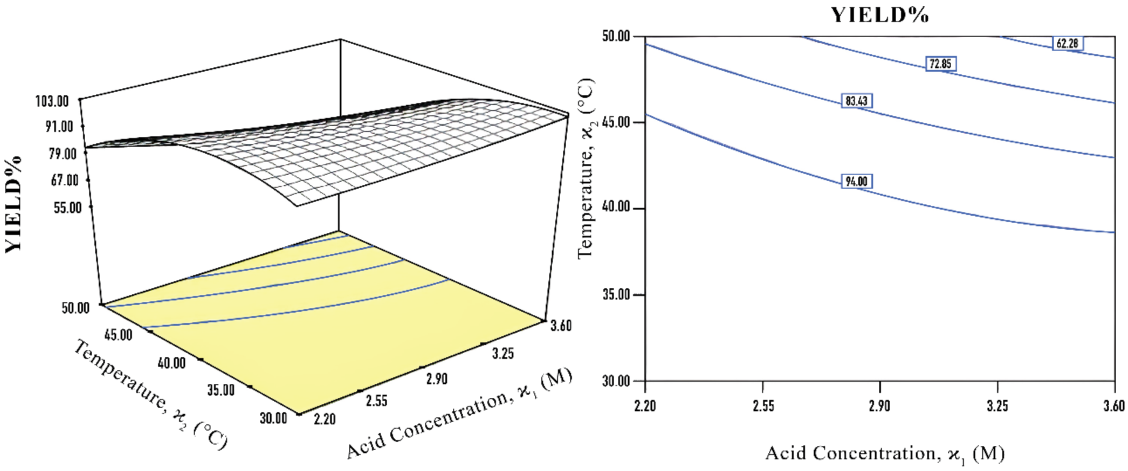

Figure S4. Response surface and contour plots illustrating the effect of acid concentration and temperature on particle size.

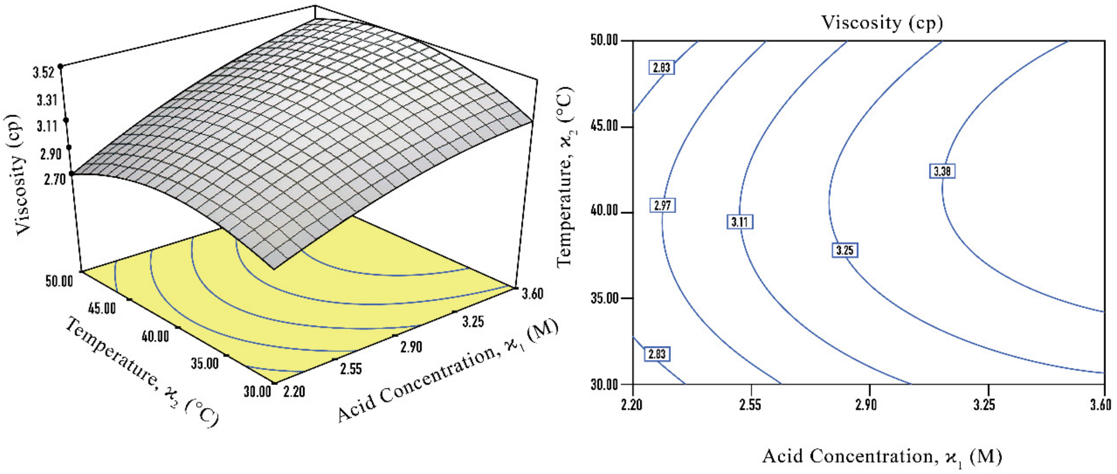

Figure S5. Response surface and contour plots demonstrating the effect of acid concentration and temperature on viscosity.

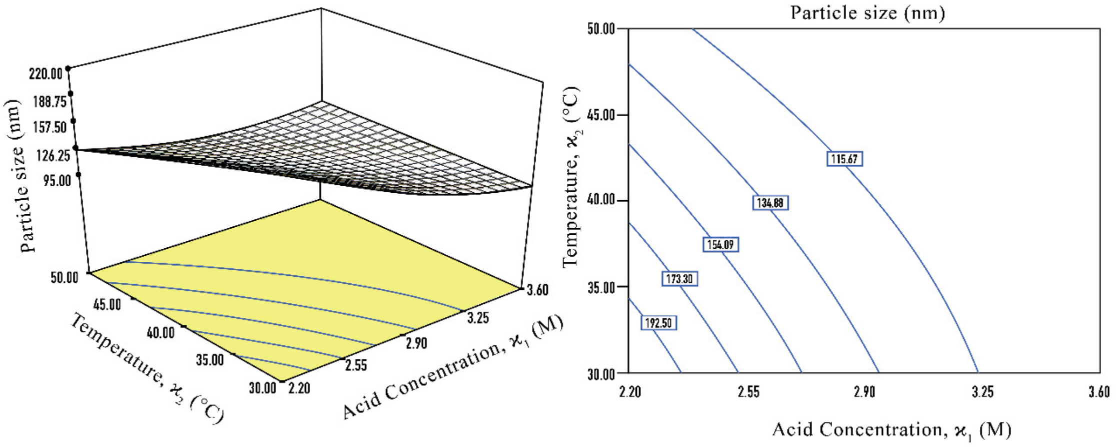

Table S3. ANOVA for response surface quadratic model for yield.

Source	Sum of squares	DF	Mean Square	F value	Prob > F	
**Model**	7737.94	9	859.77	12.69	0.0002	*significant*
$\varkappa_1$	410.55	1	410.55	6.06	0.0336	
$\varkappa_2$	3992.33	1	3992.33	58.93	<0.0001	
$\varkappa_3$	284.27	1	284.27	4.20	0.0677	
$\varkappa_1^2$	19.36	1	19.36	0.29	0.6046	
$\varkappa_2^2$	2089.96	1	2089.96	30.85	0.0002	
$\varkappa_3^2$	0.98	1	0.98	0.01	0.9065	
$\varkappa_1\varkappa_2$	433.21	1	433.21	6.39	0.0299	
$\varkappa_1\varkappa_3$	1.51	1	1.51	0.02	0.8845	
$\varkappa_2\varkappa_3$	422.68	1	422.68	6.24	0.0316	
**Residual**	677.48	10	67.75			
**Lack of fit**	557.97	5	111.59	4.67	0.0581	*not significant*

*DF = Degree of freedom that attributes to the blocks.

Table S4. ANOVA for response surface quadratic model for particle size.

Source	Sum of squares	DF	Mean Square	F value	Prob > F	
**Model**	57251.03	9	6361.23	4.31	0.0161	*significant*
x_1	17357.70	1	17357.70	11.77	0.0064	
x_2	11102.27	1	11102.27	7.53	0.0207	
x_3	5718.04	1	5718.04	3.88	0.0773	
x_1^2	4568.87	1	4568.87	3.10	0.1089	
x_2^2	17.89	1	17.89	0.01	0.9145	
x_3^2	8529.71	1	8529.71	5.78	0.0370	
x_1x_2	4027.53	1	4027.53	2.73	0.1294	
x_1x_3	549.46	1	549.46	0.37	0.5552	
x_2x_3	6412.78	1	6412.78	4.35	0.0636	
**Residual**	14746.24	10	1474.62			
**Lack of fit**	12265.99	5	2453.20	4.95	0.0521	*not significant*

*DF = Degree of freedom that attributes to blocks.

Table S5. ANOVA for response surface quadratic model for viscosity.

Source	Sum of squares	DF	Mean Square	F value	Prob > F	
**Model**	2.15	9	0.239	3.96	0.0215	*significant*
x_1	0.833	1	0.833	13.77	0.0040	
x_2	0.004	1	0.004	0.07	0.8001	
x_3	0.112	1	0.112	1.85	0.2036	
x_1^2	0.105	1	0.105	1.74	0.2166	
x_2^2	0.529	1	0.529	8.74	0.0144	
x_3^2	0.231	1	0.231	3.82	0.0790	
x_1x_2	0.031	1	0.031	0.52	0.4887	
x_1x_3	0.211	1	0.211	3.49	0.0912	
x_2x_3	0.031	1	0.031	0.52	0.4887	
**Residual**	0.605	10	0.060			
**Lack of fit**	0.337	5	0.067	1.25	0.4050	*not significant*

Table S6. Regression coefficient of each response.

Response	Std. Dev.	Mean	C.V.	R^2	Adj. R^2	Pred. R^2	Ad. Prec.
**Yield**	8.23	86.70	9.49	0.9195	0.8470	0.4714	12.941
**Particle size**	38.40	138.37	27.75	0.7952	0.6108	-0.4198	7.422
**Viscosity**	0.25	3.21	7.66	0.7807	0.5833	-0.1236	6.775
